# Accuracy prompts protect professional content moderators from the illusory truth effect

**DOI:** 10.1093/pnasnexus/pgae481

**Published:** 2024-11-19

**Authors:** Hause Lin, Marlyn Thomas Savio, Xieyining Huang, Miriah Steiger, Rachel L Guevara, Dali Szostak, Gordon Pennycook, David G Rand

**Affiliations:** Sloan School of Management, Massachusetts Institute of Technology, 100 Main St, Cambridge, MA 02142, USA; Department of Psychology, Cornell University, Uris Hall, 211, Tower Rd, Ithaca, NY 14853, USA; TaskUs, 1650 Independence Dr, New Braunfels, TX 78132, USA; TaskUs, 1650 Independence Dr, New Braunfels, TX 78132, USA; TaskUs, 1650 Independence Dr, New Braunfels, TX 78132, USA; TaskUs, 1650 Independence Dr, New Braunfels, TX 78132, USA; Google, 1600 Amphitheatre Parkway, Mountain View, CA 94043, USA; Department of Psychology, Cornell University, Uris Hall, 211, Tower Rd, Ithaca, NY 14853, USA; Hill/Levene Schools of Business, University of Regina, 3737 Wascana Parkway, Regina, SK, Canada, S4S 0A2; Sloan School of Management, Massachusetts Institute of Technology, 100 Main St, Cambridge, MA 02142, USA; Department of Brain and Cognitive Sciences, Massachusetts Institute of Technology, 43 Vassar St, Cambridge, MA 02139, USA

**Keywords:** content moderation, misinformation, trust and safety, lab-in-field experiment, illusory truth effect

## Abstract

Content moderators review problematic content for technology companies. One concern is that repeated exposure to false claims could cause moderators to come to believe the very claims they are supposed to moderate, via the “illusory truth effect.” In a first lab-in-field experiment (*N* = 199) with a global content moderation company, we found that exposure to false claims while working as moderators increased subsequent belief among (mostly Indian and Philippine) employees by 7.1%. We tested an intervention to mitigate this effect: inducing an accuracy mindset. In both general population samples (*N*_India_ = 997; *N*_Philippines_ = 1,184) and a second lab-in-field experiment with professional moderators (*N* = 239), inducing participants to consider accuracy when first exposed to the claims eliminates the negative effects of exposure on belief in falsehoods. Our results show that the illusory truth effect and the protective power of an accuracy mindset generalize to non-Western populations and professional moderators.

**Significance Statement**

This research investigates the impact of repeated exposure to misinformation on professional content moderators, a critical but often overlooked group in digital ecosystems. Across three field and survey experiments conducted in a global context, we find that even content moderators who regularly evaluate problematic content are susceptible to the “illusory truth effect”—the phenomenon where repeated exposure increases belief in the exposed claims. Importantly, we provide evidence that an accuracy-focused mindset protects against the effect for false claims, thus providing evidence for a simple and practical solution to this problem. These findings underscore the importance of strategic interventions in content moderation processes to maintain the integrity of online platforms and combat misinformation’s pervasive influence, ensuring a healthier digital environment for all.

## Introduction

Content moderation has become one of the most crucial jobs created by the internet economy (1). With the expansion of online platforms, the number of human moderators has grown correspondingly and is now estimated to number in the hundreds of thousands worldwide (2). Technology companies rely on human content moderators—typically located in non-Western countries—to identify problematic content on their platforms. Thus, content moderators play a key role in making the internet a safer space for everyone. Yet because they regularly review problematic content (3), there is reason to expect that they may become more likely to think such content is accurate due to the “illusory truth effect” (also known as the repetition-induced truth effect or repeated exposure effect) (4–6). Here, we investigate through experimental tasks whether content moderators indeed believe false claims more after being exposed to them. We also examine whether encouraging an accuracy mindset at exposure reduces this effect.

A large body of literature from behavioral and cognitive science demonstrates that repeated exposure to claims increases their perceived accuracy (4, 5), regardless of whether they are actually true or false (7). This effect has been demonstrated using trivia statements (8) and false content that contradicts prior knowledge (9), but also for moral transgressions (10) and fake news posts taken from social media (11, 12), and has been found to be equally strong for people who are more analytical, cognitive sophisticated, or intelligent (13).

Although no study has investigated professional content moderators’ susceptibility to the illusory truth effect, a Verge article reported that moderators started embracing fringe and often hate-mongering conspiracy theories that they were supposed to moderate and would dismiss under normal circumstances (3). For example, former moderators reported that the kinds of content they had been exposed to while moderating had gradually made them doubt aspects of the Holocaust and disbelieve 9/11 was a terrorist attack. These reports highlight the need to examine whether repeated exposure to false, problematic content indeed causally increases their perceived accuracy among content moderators. If so, there are important consequences not just for the moderators themselves (3, 14) but also for society at large (1). Their ability to evaluate the veracity of repeated claims may be compromised, which could lead to the inadvertent spread of misinformation, as moderators start believing and thus failing to flag repeated false claims, and interventions will be necessary to mitigate these effects.

However, the nature of the moderators’ jobs might actually protect them against the illusory truth effect. Because their job requires moderators to regularly review content and decide whether to remove it based on objective criteria, they might be keenly attuned to the veracity of the content they are reviewing. Such an accuracy-focused mindset has been shown to buffer experimental participants against the illusory truth effect on false claims, for both trivia statements (15) and news headlines (11). Thus, if moderators are considering accuracy by default during exposure, repetition might not cause moderators to actually come to believe false claims.

In addition, content moderators are often located in non-Western parts of the world, especially in India and the Philippines (1). Surprisingly, there is a lack of empirical evidence investigating whether the illusory truth effect replicates in non-Western countries (4), where cultural differences in cognitive processing could influence how repetition affects perceived truthfulness. For example, non-Western cultures often emphasize holistic (vs. analytic) thinking (16), and holistic thinkers might be less influenced by repetition because they rely more on contextual cues and relationships between objects rather than the frequency of exposure. Examining the generalizability of the illusory truth effect to non-Western and content moderation contexts can therefore provide important insights (17, 18) that can improve theorizing on the phenomenon and interventions designed to mitigate it.

Here, we partnered with TaskUs, a global business process outsourcing service provider content moderation company that provides moderation for major technology companies, to investigate whether the illusory truth effect occurs among professional content moderators, based in India and the Philippines. All the experiments reported below were preregistered and used stimuli (48 headlines: 24 true and 24 false) that were related to the COVID-19 pandemic, which was an important and globally relevant problem when the experiments were conducted (19, 20).

**Fig. 1.**
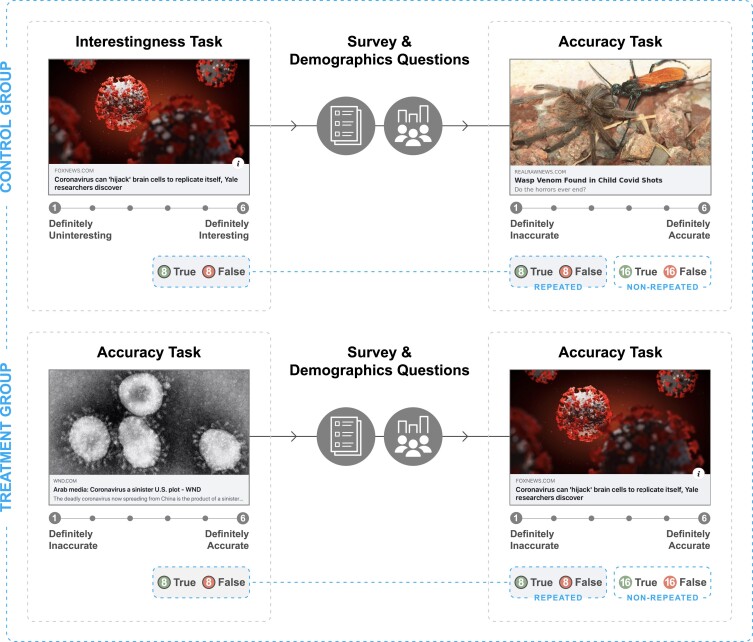
Study design. The first experiment included only the control condition (Top). The next two experiments included both the control and treatment conditions. In all experiments, participants first rated the interestingness or accuracy of 16 headlines (8 true and 8 false), completed a distractor task (e.g. questionnaires), and then rated the accuracy of 48 headlines (the 16 repeated headlines from the interestingness task plus 32 new, nonrepeated, headlines).

In a lab-in-field experiment (Fig. 1 Top panel only), we demonstrate that professional content moderators do indeed come to believe false claims more after being exposed to them. We then test whether it is possible to protect against such illusory truth effects for content moderators by inducing them to consider accuracy when they are initially exposed to the claims (Fig. 1). In a pair of survey experiments conducted with general population samples in India and the Philippines, we replicate the repetition effect as well as previous findings that prompting participants to consider headlines’ accuracy when being initially exposed to them reduces the effect of exposure on falsehoods (11, 15). Finally, in a second experiment with content moderators, we demonstrate that inducing an accuracy mindset also successfully protects them against exposure increasing belief in false claims.

Overall, our preregistered lab-in-field and survey experiments, conducted in non-Western and content moderation contexts, highlight the illusory truth effect’s robustness. This effect, previously studied primarily in Western laboratory contexts, persists among non-Western populations and even professional content moderators, with similar effect sizes across these contexts. Importantly, we show that the ameliorative power of prompting people to consider accuracy when evaluating claims also generalizes to non-Western populations and professional content moderators. Thus, inducing an accuracy mindset is a promising approach for mitigating the illusory truth effect among professional content moderators.

## Results

### Do professional fact-checkers judge repeated content as more accurate?

In a first preregistered lab-in-field experiment, 199 professional content moderators were asked to complete a rating task during their workday. They first rated 16 COVID-19 news headlines on their interestingness, then completed an intervening distractor task, and finally rated the accuracy of the 16 headlines from the first rating task (“repeated” headlines) along with 32 additional new (“nonrepeated”) COVID-19 news headlines (see Fig. 1 Top panel).

**Fig. 2.**
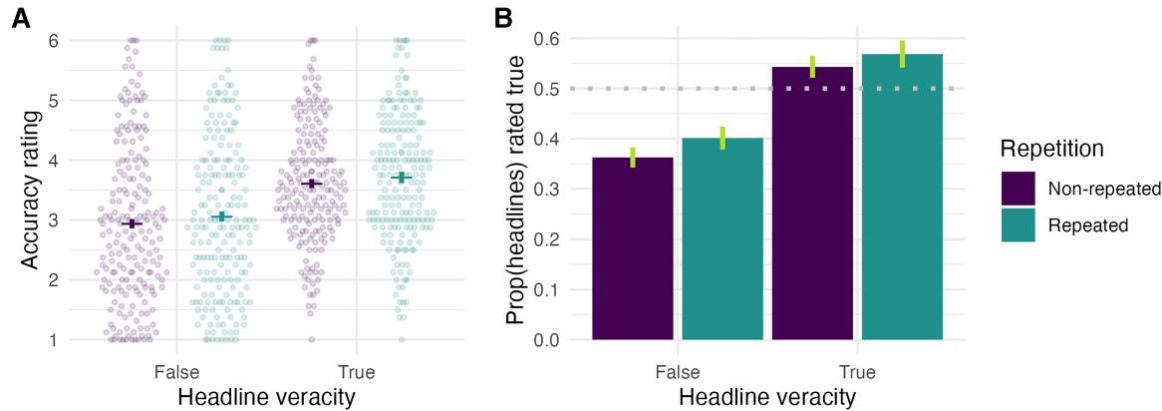
Exposure increases subsequent belief in headlines regardless of their veracity. A) Accuracy ratings (each dot is one moderator’s mean rating) and B) proportion of headlines rated as true (i.e. above accuracy scale midpoint) by headline veracity and repetition. Raw means are shown, and vertical lines indicate 95% within-subject CIs.

As shown in Fig. 2, content moderators were susceptible to repetition effects. The moderators judged repeated headlines as more accurate than nonrepeated headlines (*b* = 0.10 [0.04, 0.15]; Fig. 2A). While they also judged true headlines as more accurate than false headlines (*b* = 0.66 [0.45, 0.87]), there was no evidence of a meaningful interaction between veracity and repetition (*b* = −0.03 [−0.13, 0.07]), such that the repetition effect was present to a similar extent for both true and false headlines. To aid interpretation of these effect sizes (Fig. 2B), we discretize accuracy ratings into true (above scale midpoint) and false (below scale midpoint), and note that moderators rated 45.3% of the new headlines as true, compared with 48.5% of the repeated headlines (i.e. 7.1% increase).

The results and effect sizes were almost identical after controlling for several covariates (age, gender, disposition for analytic thinking, COVID-19 concern, socioeconomic status, attention, education, belief in God, and job experience). There were clear effects of headline veracity (*b* = 0.65 [0.45, 0.85]) and repetition (*b* = 0.10 [0.04, 0.15]) but no meaningful veracity–repetition interaction (*b* = −0.02 [−0.13, 0.08]). We also found meaningful interactions between headline veracity and (i) disposition for analytic thinking as measured by the Actively Open-minded Thinking questionnaire (21) (*b* = 0.33 [0.21, 0.46]) and (ii) COVID-19 concern (*b* = 0.23 [0.10, 0.36]). That is, moderators who were more analytic were better at discerning true from false headlines, in line with prior findings (e.g. (20)), as were moderators who were more concerned about COVID-19. Finally, there was a three-way interaction between headline veracity, repetition, and COVID-19 concern (*b* = 0.16 [0.04, 0.27]), such that moderators who were concerned about COVID-19 were particularly better at discerning true from false headlines for repeated headlines. Note, however, these interaction tests were not preregistered and posttreatment bias might be an issue because the individual difference variables/moderators were measured posttreatment (22) (see Table S1 for regression table).

### Protecting against exposure effects by inducing an accuracy mindset in general population samples

Having shown that professional content moderators are susceptible to illusory truth effects, we then examined whether inducing an accuracy mindset when being initially exposed to headlines protects against such pernicious effects (11, 15). To our knowledge, there is no empirical evidence for either the baseline repetition effect or the protective effect of accuracy mindset in cultures in which the moderators are primarily based (or in any non-US or Western countries). Therefore, before conducting another experiment inducing an accuracy mindset among content moderators, we first conducted large preregistered survey experiments (using COVID-19 news headlines) with general population samples in India (*N* = 997) and the Philippines (*N* = 1,184) to examine cross-cultural generalizability.

**Fig. 3.**
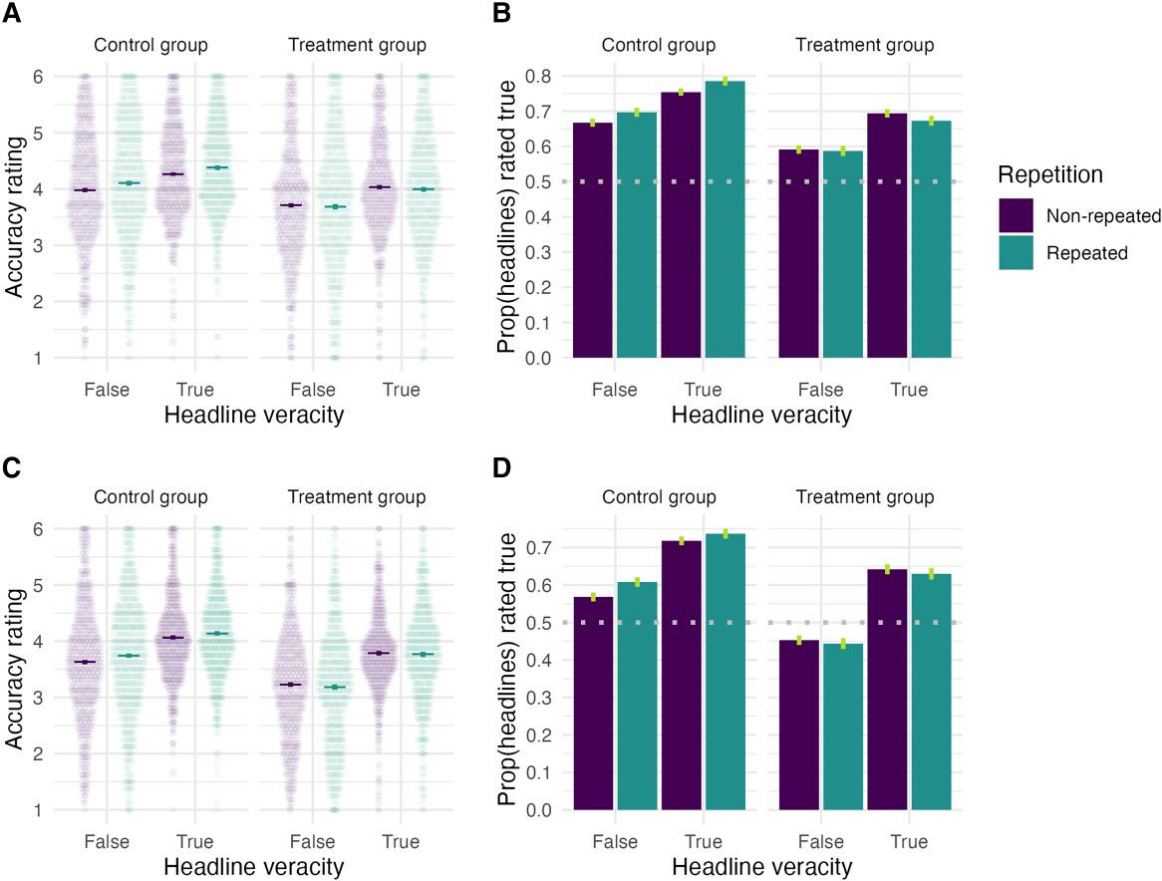
Effect of experimental condition, headline veracity, and repetition on accuracy judgments for Indian and Philippine participants. A) Indian participants’ accuracy ratings (each dot is one participant's mean rating) and B) proportion of headlines rated as true (i.e. above accuracy scale midpoint) by experimental condition, headline veracity, and repetition. C) Philippine participants’ accuracy ratings and D) proportion of headlines rated as true (i.e. above accuracy scale midpoint) by experimental condition, headline veracity, and repetition. Raw means are shown, and vertical lines indicate 95% within-subject CIs.

Following our preregistration, we first ask whether the illusory truth effect is observed on false headlines in the control condition where participants were asked to judge the interestingness of the headlines during the exposure phase. We find that the illusory truth effect robustly replicated cross-culturally (Fig. 3): Repeated false headlines in the control condition were judged as more accurate than nonrepeated headlines in both countries (*b*_India_ = 0.11 [0.06, 0.17], 4.4% increase; *b*_Philippines_ = 0.12 [0.08, 0.15], 7.0% increase), and the effects were similar in magnitude relative to those observed among the content moderators (i.e. *b* = 0.10 [0.04, 0.15], 7.1% increase).

Next, we ask whether the illusory truth effect for false headlines is reduced by an intervention that directs attention to accuracy—specifically, by asking participants to judge the accuracy (rather than interestingness) of the headlines during the exposure phase. Indeed, we find robust negative repetition–condition interactions in both countries (*b*_India_ = −0.14 [−0.22, −0.07]; *b*_Philippines_ = −0.16 [−0.22, −0.11]), such that the accuracy prompt treatment entirely eliminated—and actually *reversed—*repetition effects for false headlines (Fig. 3): A post hoc analysis found that participants in the treatment condition judged repeated false headlines as *less* accurate than nonrepeated false headlines (*b*_India_ = −0.03 [−0.08, 0.01]; *b*_Philippines_ = −0.05 [−0.09, −0.01]).

The effect sizes for the repetition and repetition–condition interaction effects were similar after controlling for several covariates (age, gender, education, income, disposition for analytic thinking, COVID-19 concern, conspiracy mentality; doubly robust average treatment effects from causal forests: *b*_India_ = −0.15 [−0.22, −0.08]; *b*_Philippines_ = −0.16 [−0.22, −0.10]). Interestingly, we found interactions such that the baseline repetition effect was reduced for participants with higher income in India (*b* = −0.06 [−0.11, −0.01]) and for participants with greater disposition for analytic thinking in the Philippines (*b* = −0.03 [−0.06, 0.00]). The treatment was also more effective at buffering the repetition effect among more educated Indian participants (repetition–condition–education interaction effect: *b* = −0.10 [−0.18, −0.02]). See Tables S2 and S3 for regression tables; note that as in the first study, these interaction effect tests were not preregistered and all moderators were measured posttreatment.

We now shift from considering only the false statements to asking whether the baseline illusory truth effect was moderated by headline veracity. Because the repetition–condition interaction effect reported above indicates that the repetition effect has been attenuated in the treatment condition, we therefore—following our preregistration—focus on only participants in the control condition for this analysis (*n*_India_ = 512; *n*_Philippines_ = 610). We find that participants in both countries judged true headlines as more accurate than false ones (*b*_India_ = 0.28 [0.14, 0.42]; *b*_Philippines_ = 0.42 [0.23, 0.61]), judged repeated headlines (true and false ones) as more accurate than nonrepeated ones (*b*_India_ = 0.11 [0.07, 0.16]; *b*_Philippines_ = 0.10 [0.06, 0.14]), and—critically—the repetition effect was similar for both true and false headlines (i.e. no veracity–repetition interaction effect: *b*_India_ = −0.01 [−0.09, 0.07]; *b*_Philippines_ = −0.03 [−0.10, 0.04]). These effects were robust after controlling for the same covariates as above, and the repetition effect was weaker among higher-income Indian participants (*b* = −0.04 [−0.07, 0.00]; see Tables S4 and S5 for regression tables).

### Inducing an accuracy mindset among professional content moderators

Finally, we conducted a second preregistered experiment with TaskUs content moderators (*N* = 239) to evaluate the effectiveness of the accuracy prompt intervention in mitigating repetition effects for false claims among professional moderators. This experiment used the same design as the general population sample experiments described above, but was delivered as a lab-in-field experiment with TaskUs.

**Fig. 4.**
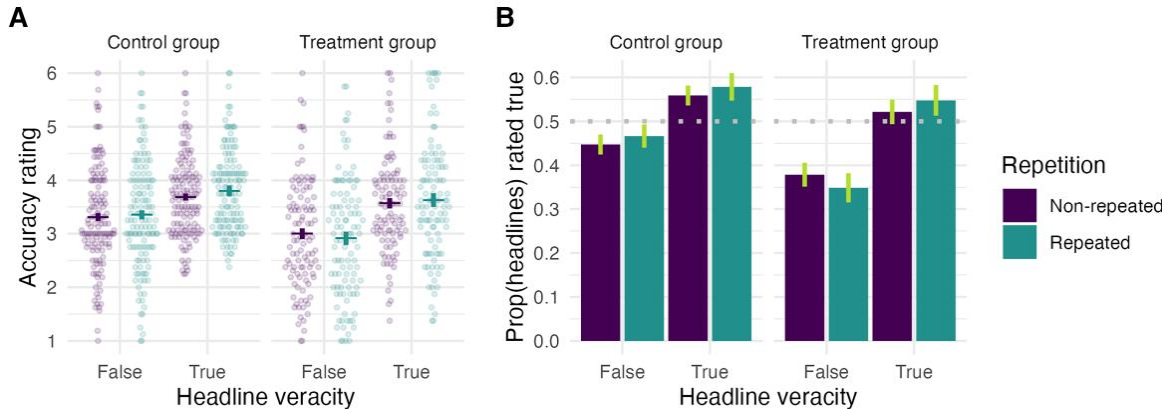
Effect of experimental condition, headline veracity, and repetition on accuracy judgments for professional content moderators. A) Accuracy ratings (each dot is one moderator’s mean rating) and B) proportion of headlines rated as true (i.e. above accuracy scale midpoint) by experimental condition, headline veracity, and repetition. Raw means are shown, and vertical lines indicate 95% within-subject CIs.

As expected, we replicated the repetition effect for false headlines, such that repeated false headlines were judged as more accurate than nonrepeated false headlines (*b* = 0.09 [0.01, 0.18]; Fig. 4). Most importantly, the moderators in the treatment condition (who rated headline accuracy during initial exposure) judged repeated false headlines as less accurate than those in the control condition (who rated headline interestingness during initial exposure) (repetition–condition interaction effect: *b* = −0.15 [−0.28, −0.02]; Fig. 4). The interaction effect was similar when controlling for different covariates in the same regression model (*b* = −0.19 [−0.35, −0.03]) or estimated using doubly robust methods (*b* = −0.13 [−0.26, −0.01]). There was also some evidence that the intervention was more effective for male content moderators (repetition–condition–gender interaction effect: *b* = −0.18 [−0.34, −0.02]; see Table S6 for regression table; note again that these interaction effect tests were not preregistered and all moderators were measured posttreatment). Overall, these findings provide evidence that the accuracy prompt intervention successfully protected content moderators against the repetition effect for false claims.

We then ask whether the illusory truth effect was moderated by headline veracity. Because the repetition effect was attenuated in the treatment condition, we again—following our preregistration—focused only on moderators in the control condition for this analysis (*n* = 134). We find that they judged true headlines as more accurate than false ones (*b* = 0.40 [0.21, 0.58]; Fig. 4). The overall repetition effect (for true and false headlines) was weaker relative to the effects reported in the previous studies (*b* = 0.06 [−0.01, 0.12]), and the effect was not moderated by headline veracity (*b* = −0.05 [−0.17, 0.06]). The results were similar after controlling for several covariates (repetition effect: *b* = 0.08 [0.01, 0.15]), and there was some evidence that the repetition effect was weaker for older moderators (repetition–age interaction effect: *b* = −0.07 [−0.14, −0.01]; see Table S7 for regression table).

## Discussion

Content moderators play a crucial role in ensuring the safety and integrity of online platforms. In the cross-cultural and survey experiments reported here, we find evidence showing that the illusory truth effect is not idiosyncratic to Western populations (4, 23) and replicates even among content moderators whose primary role is evaluating content in potentially high-stakes real-world contexts (1, 3). They judged news headlines they had been previously exposed to as more accurate than novel headlines, regardless of whether the headlines were actually true or false. That is, despite their professional training, content moderators are susceptible to cognitive biases that can influence their judgments, consistent with reports suggesting that moderators may not always make moderation decisions primarily with accuracy in mind when they are on the job (1, 3).

Our results demonstrate the illusory truth effect in a population whose behavior has direct effects on the safety and integrity of online platforms. They highlight the potential negative consequences of repeated exposure to problematic content, both for the content moderators themselves and for the health of the internet more broadly (24). The nature of content moderators’ work—identifying problematic content on platforms—makes them more likely to be repeatedly exposed to falsehoods (3). Exposure effects have been found to compound over multiple exposures (12), and these effects may cause moderators to become less effective at moderating content over time (at least in terms of accuracy). These negative compounding effects could lead to lower-quality training data for machine algorithms designed to flag problematic content for others to review (25).

Our studies suggest that the illusory truth effect may, in the long term, compromise the safety and integrity of online platforms and that better training, interventions, or policies might be necessary to mitigate the effect. Importantly, simple and effective evidence-based solutions that prompt moderators to attend to accuracy could address these issues (20, 26–28).

Our findings also highlight the need to examine the different consequences of content moderation (in addition to the emotional consequences (14, 29, 30); see also (31, 32)). Future work should also investigate the illusory truth effect among another important population: professional fact-checkers (33, 34) who play critical roles in reviewing content for technology platforms.

Importantly, however, our findings not only identify the potential harm of exposure, but also demonstrate the effectiveness of a simple solution. We find that exposure's pernicious effects can be successfully mitigated by prompting moderators to consider accuracy when evaluating the claims they encounter. Our results demonstrate the benefits of keeping accuracy salient for moderators while they evaluate content—for example by occasionally asking them to judge the accuracy of content they see even if that is not their primary moderation goal.

Beyond these important implications for online trust and safety, our work also has theoretical implications. By providing evidence from cross-cultural and content moderation contexts, our results highlight the robustness and pervasiveness of the illusory truth effect (4–6). Furthermore, we find little evidence that cognitive or social factors (e.g. analytic thinking) reliably protect against the illusory truth effect, echoing prior null moderation findings (13). On the positive side, however, there were also no consistent moderators of the effectiveness of the accuracy prompt intervention. The fact that this treatment effect was not robustly moderated by any individual differences is promising because it suggests the intervention may be widely effective across individuals, cultures, and contexts (17). These results are also consistent with the finding that accuracy prompts reduce misinformation sharing in cross-cultural survey experiments and field experiments (20, 27, 28).

In sum, the cross-cultural and lab-in-field experiments reported here identify the illusory truth effect as a pernicious effect that should be addressed to combat misinformation. Developing interventions that are easy for companies to test and scale up will be crucial to mitigating the illusory truth effect among the most “at-risk” populations like content moderators—because they are the most likely to be exposed to misinformation and their susceptibility or resistance to the effect can have far-reaching downstream effects on the health of the internet.

## Materials and methods

All methods were approved by the MIT Institutional Review Board, and informed consent was obtained from all participants. We preregistered the data collection and analytic plans for all three studies. Unless stated as post hoc, all analyses are preregistered. In all studies, we preregistered using Bayesian sampling instead of frequentist methods because, for mixed models with crossed random effects like in our studies, the models tend not to converge when fitted using frequentist estimation methods (and simply dropping terms to reduce model complexity until the model converges is suboptimal because the full desired model specification is not used). Moreover, the estimates and 95% CIs/credible intervals from Bayesian and frequentist methods are generally identical, so the estimates from our Bayesian mixed modeling approach correspond to those from frequentist modeling approaches. Our preregistration, materials, and analysis code are available on Open Science Framework (OSF): https://osf.io/t47am/.

### Study 1: Content moderator field experiment 1

We designed the study with TaskUs, a global content moderation company, who then recruited their content moderator teammates to complete the preregistered study (https://aspredicted.org/jyd_nx5). The moderators were based in South and Southeast Asia (predominantly India and the Philippines) and were trained to moderate English content. Content moderators with at least a 3-month tenure at the company were eligible to participate during their work shift. Of the 239 moderators recruited into the study, 199 (83%) completed it. Because the study design was entirely within-subjects, attrition does not pose a problem for causal inference regarding the effect of repetition.

The task used 48 COVID-19 headlines (veracity: 24 true and 24 false), which were “up to date” or relevant when the study was run. The false headlines were found using popular fact-checking sites (e.g. snopes.com), whereas the true headlines came from reputable mainstream news sources.

In section one of the study (Fig. 1 Top panel only), each moderator rated a random subset of 16 headlines (8 true and 8 false) on how interesting the claim in each headline was (6-point scale; 1: definitely uninteresting and 6: definitely interesting). They then completed the Actively Open-minded Thinking questionnaire (an analytic thinking disposition measure) (21), single-item COVID-19 concern question (“How concerned are you about COVID-19?”; 6-point scale; 1: not at all and 6: extremely), single-item belief in God question (“How much would you say you believe in God or Gods?”; 7-point scale; 1: not at all and 7: very much), and various demographic measures (age, gender, education, socioeconomic status, ethnicity; note that job experience was provided by the company after the study ended). Then, the moderators completed section two where they rated how accurate the claim in each headline was (6-point scale; 1: definitely inaccurate and 6: definitely accurate). They rated all 48 headlines in this section: 16 were “repeated” because the moderators had already seen and rated these headlines (on interestingness) in the previous section, but 32 were “nonrepeated” because they had not been shown in the previous section.

We also included two attention check questions (one presented before section one and another before the COVID-19 concern question). We did not exclude moderators who failed the checks but included mean accuracy across both questions as a covariate in our analysis.

We fit Bayesian linear mixed-effects models to predict accuracy ratings as a function of headline veracity (true [0.5] or false [−0.5]) and repetition (repeated [0.5] or nonrepeated [−0.5]) and their interactions and modeled varying intercepts and slopes: R syntax: accuracy ∼ veracity × repetition + (veracity × repetition | id) + (1 | headline). We also performed robustness checks by controlling for covariates (*z*-scored). Two-way interaction models: accuracy ∼ veracity × (repetition + covariate_1_ + … + covariate_n_ | id) + (1 | headline); three-way interaction models: accuracy ∼ veracity × repetition × (covariate_1_ + … + covariate_n_ | id) + (1 | headline) (see Table S1).

### Study 2: Cross-cultural Indian and Philippine experiments

We recruited participants from India (*N* = 997; *n*_treatment_ = 485) and the Philippines (*N* = 1,184; *n*_treatment_ = 574) from Lucid (preregistration: https://aspredicted.org/RYZ_Q4R). Participants were randomly assigned to either the control or treatment condition (Fig. 1). The task used 48 COVID-19 headlines (24 true and 24 false) that were “up to date” or relevant when the study was run. The experimental procedure was similar to study 1's. In section one, participants rated a random subset of 16 headlines (8 true and 8 false) on how interesting (control condition) or accurate (treatment condition) the claim in each headline was (6-point scale). They then completed questionnaires (i.e. Actively Open-minded Thinking, COVID-19 concern, conspiracy mentality (35)) and various demographic measures (e.g. age, gender, education, and income). Finally, participants in both conditions completed the same section two where they rated how accurate the claim in each headline was (48 headlines in total: 16 were repeated and 32 were nonrepeated [not shown in previous section]).

We fitted two sets of Bayesian linear mixed-effects models (see preregistration). First, we tested whether inducing the accuracy mindset in the treatment condition attenuates the repetition effect for false headlines by modeling accuracy ratings as a function of repetition (repeated [0.5] or nonrepeated [−0.5]), condition (control [0] or treatment [1]), and their interactions and modeled varying intercepts and slopes. As with study 1, we perform robustness checks by controlling for covariates (see Tables S2 and S3).

Second, we tested whether the repetition effect differed for true and false headlines. As preregistered, we included only participants in the *control* condition because the presence of repetition–condition interaction effects implies that the repetition effect—when including participants in both the control and treatment conditions—is biased because it is attenuated in the treatment condition. We therefore fitted the models to only control participants to examine whether accuracy ratings vary as a function of repetition (repeated [0.5] or nonrepeated [−0.5]), veracity (false [−0.5] or true [0.5]), and their interaction. Similar to the model above, we also perform robustness checks by controlling for covariates (see Tables S4 and S5).

We did not find evidence for differential attrition at any stage of the experiment (χ^2^ < 1.84, df = 1, ps > 0.200 for all). Nevertheless, we checked whether the conditions differed on any covariates and found that in the India experiment, participants in the treatment group reported lower belief in God (*P* = 0.021; likely a false positive due to multiple testing) and scored higher on the Actively Open-minded Thinking measure (*P* = 0.006). Given the lack of evidence of differential attrition, the difference in Actively Open-minded Thinking is likely due to the treatment itself changing responses, since the individual differences were measured posttreatment (i.e. posttreatment bias (22)) (Fig. 1). We also show that our treatment effects are robust: They survive when controlling for all covariates in the regression models (see Tables S2 and S3) and when fitting a causal forest where doubly robust treatment effects are estimated (36, 37).

### Study 3: Content moderator lab-in-field experiment 2

We designed the second experiment with the same content moderation company, TaskUs (preregistration: https://aspredicted.org/FZP_9TX). They recruited TaskUs content moderators who had not participated in study 1 to complete this second experiment (*N* = 239; *n*_treatment_ = 105). The design was the same as study 2 above in which the moderators were randomly assigned to the control or treatment condition (Fig. 1). The task used 48 COVID-19 headlines (24 true and 24 false) that were “up to date” or relevant when the study was run. The design and procedure were identical to study 2. We administered the same questionnaires and demographics measures in the first experiment. We also included two attention check questions (one presented before section one and another before the COVID-19 concern question). We did not exclude moderators who failed the checks but included mean accuracy across both questions as a covariate in our analysis. The preregistered analysis plan and modeling approach are similar to study 2's plan—we fitted the two sets of Bayesian linear mixed-effects models described above.

Of the 289 moderators recruited into the study, 239 (83%) completed it. Due to the limitations of the survey platform used for this experiment, we do not know which condition the 50 attriters were assigned to. As with the general population samples in study 2, the moderators in the control (*n* = 134) and treatment (*n* = 105) conditions did not significantly differ on any covariate, except that—as in the general population experiment—the moderators in the treatment condition scored significantly higher on the Actively Open-minded Thinking measure (*P* = 0.006). Given that the same effect was observed in the general population experiment where there was no evidence of differential attrition, this difference is likely driven by the treatment changing responses to the Actively Open-minded Thinking measure, rather than differential attrition. Finally, two robustness checks provide additional evidence that the treatment effects were unlikely to be driven by differential attrition: The treatment effects survive when controlling for all covariates in the regression models (see Table S6) and when fitting a causal forest where doubly robust treatment effects are estimated (36, 37).

## Supplementary Material

pgae481_Supplementary_Data

## Data Availability

Full reproduction materials including data and analysis code are accessible at: https://osf.io/t47am/.

## Preprints

This manuscript was posted on a preprint: https://osf.io/preprints/psyarxiv/gswm6.
