# Supplementary material for: Accuracy prompts protect professional content moderators from the illusory truth effect
Source: PNAS Nexus. 2024 Nov 19;3(11):pgae481. doi: 10.1093/pnasnexus/pgae481 (PMC11574866; doi:10.1093/pnasnexus/pgae481)
Supplement: pgae481_Supplementary_Data [file pgae481_supplementary_data.pdf]

PNAS nexus

Supplementary Information for
Accuracy prompts protect professional content moderators from the illusory truth effect

Hause Lin[1,2], Marlyn Thomas Savio[3], Xieyining Huang[3], Miriah Steiger[3], Rachel L. Guevara[3], Dali Szostak[4], Gordon Pennycook[2,5], David G. Rand[1,6]
Hause Lin
Email: hauselin@gmail.com

This PDF file includes:

Tables S1 to S7

Supplementary Table 1. Content moderator field experiment 1

Parameter	Estimate
Intercept	3.33 (0.08) [3.17, 3.49]
veracityC	0.65 (0.10) [0.45, 0.85]
repetitionC	0.10 (0.03) [0.04, 0.15]
ageZ	-0.08 (0.07) [-0.21, 0.06]
genderZ	0.13 (0.07) [-0.01, 0.27]
aotZ	-0.46 (0.07) [-0.60, -0.31]
covidconcernZ	-0.10 (0.08) [-0.26, 0.06]
sesZ	0.14 (0.07) [0.01, 0.28]
attentionZ	-0.13 (0.07) [-0.28, 0.01]
educationZ	-0.04 (0.07) [-0.18, 0.10]
godZ	0.16 (0.07) [0.02, 0.31]
hireZ	0.12 (0.07) [-0.01, 0.26]
veracityC × repetitionC	-0.02 (0.05) [-0.12, 0.08]
veracityC × ageZ	0.03 (0.06) [-0.09, 0.15]
veracityC × genderZ	-0.08 (0.06) [-0.21, 0.04]
veracityC × aotZ	0.33 (0.07) [0.20, 0.46]
veracityC × covidconcernZ	0.23 (0.07) [0.10, 0.36]
veracityC × sesZ	-0.07 (0.06) [-0.19, 0.05]
veracityC × attentionZ	0.07 (0.06) [-0.06, 0.19]
veracityC × educationZ	0.08 (0.06) [-0.04, 0.20]
veracityC × godZ	-0.04 (0.06) [-0.17, 0.08]
veracityC × hireZ	-0.02 (0.06) [-0.14, 0.10]
repetitionC × ageZ	0.02 (0.03) [-0.04, 0.07]
repetitionC × genderZ	0.02 (0.03) [-0.04, 0.07]
repetitionC × aotZ	0.03 (0.03) [-0.02, 0.09]
repetitionC × covidconcernZ	0.03 (0.03) [-0.03, 0.09]
repetitionC × sesZ	-0.05 (0.03) [-0.10, 0.01]
repetitionC × attentionZ	0.00 (0.03) [-0.06, 0.05]
repetitionC × educationZ	0.00 (0.03) [-0.05, 0.06]
repetitionC × godZ	0.00 (0.03) [-0.06, 0.06]
repetitionC × hireZ	0.01 (0.03) [-0.04, 0.06]
veracityC × repetitionC × ageZ	0.04 (0.05) [-0.07, 0.14]
veracityC × repetitionC × genderZ	0.04 (0.05) [-0.06, 0.15]
veracityC × repetitionC × aotZ	0.02 (0.06) [-0.09, 0.13]
veracityC × repetitionC × covidconcernZ	0.16 (0.06) [0.04, 0.27]
veracityC × repetitionC × sesZ	-0.07 (0.05) [-0.17, 0.04]
veracityC × repetitionC × attentionZ	-0.07 (0.06) [-0.19, 0.04]
veracityC × repetitionC × educationZ	-0.07 (0.05) [-0.17, 0.04]
veracityC × repetitionC × godZ	-0.04 (0.06) [-0.16, 0.07]
veracityC × repetitionC × hireZ	0.06 (0.05) [-0.05, 0.16]

Standard deviations and 95% intervals of posterior distributions are shown.

Supplementary Table 2. India experiment (control and treatment groups)

Parameter	Estimate
Intercept	3.84 (0.08) [3.69, 3.99]
repetitionc	0.12 (0.03) [0.06, 0.18]
conditiond	-0.24 (0.07) [-0.38, -0.11]
age	-0.16 (0.04) [-0.25, -0.07]
gender	0.03 (0.04) [-0.05, 0.11]
education	0.06 (0.04) [-0.03, 0.15]
income	0.04 (0.04) [-0.05, 0.12]
aot	-0.47 (0.05) [-0.56, -0.38]
covid_concern	0.10 (0.04) [0.02, 0.18]
conspiracy	0.11 (0.04) [0.03, 0.20]
repetitionc × conditiond	-0.13 (0.04) [-0.21, -0.05]
repetitionc × age	0.04 (0.03) [-0.02, 0.09]
repetitionc × gender	0.02 (0.03) [-0.03, 0.07]
repetitionc × education	0.06 (0.03) [0.00, 0.11]
repetitionc × income	-0.06 (0.03) [-0.11, -0.01]
repetitionc × aot	0.01 (0.03) [-0.05, 0.07]
repetitionc × covid_concern	0.03 (0.03) [-0.02, 0.08]
repetitionc × conspiracy	0.02 (0.03) [-0.03, 0.08]
conditiond × age	-0.01 (0.06) [-0.13, 0.11]
conditiond × gender	0.01 (0.06) [-0.11, 0.13]
conditiond × education	0.06 (0.06) [-0.06, 0.18]
conditiond × income	-0.01 (0.06) [-0.12, 0.11]
conditiond × aot	0.07 (0.06) [-0.06, 0.19]
conditiond × covid_concern	0.04 (0.06) [-0.08, 0.16]
conditiond × conspiracy	0.05 (0.06) [-0.07, 0.16]
repetitionc × conditiond × age	-0.01 (0.04) [-0.09, 0.07]
repetitionc × conditiond × gender	-0.01 (0.04) [-0.09, 0.06]
repetitionc × conditiond × education	-0.10 (0.04) [-0.18, -0.02]
repetitionc × conditiond × income	0.01 (0.04) [-0.06, 0.09]
repetitionc × conditiond × aot	-0.06 (0.04) [-0.14, 0.02]
repetitionc × conditiond × covid_concern	-0.05 (0.04) [-0.12, 0.03]
repetitionc × conditiond × conspiracy	-0.02 (0.04) [-0.09, 0.06]

Standard deviations and 95% intervals of posterior distributions are shown. Only false headlines are included.

Supplementary Table 3. Philippines experiment (control and treatment groups)

Parameter	Estimate
Intercept	3.75 (0.09) [3.58, 3.93]
repetitionc	0.13 (0.02) [0.08, 0.17]
conditiond	-0.44 (0.06) [-0.56, -0.31]
age	-0.10 (0.04) [-0.17, -0.02]
gender	0.11 (0.04) [0.04, 0.19]
education	-0.09 (0.04) [-0.17, -0.01]
income	0.01 (0.04) [-0.07, 0.09]
aot	-0.45 (0.04) [-0.53, -0.38]
covid_concern	0.05 (0.04) [-0.02, 0.13]
conspiracy	0.20 (0.04) [0.13, 0.28]
repetitionc × conditiond	-0.16 (0.03) [-0.22, -0.09]
repetitionc × age	-0.01 (0.02) [-0.05, 0.03]
repetitionc × gender	0.01 (0.02) [-0.03, 0.05]
repetitionc × education	0.00 (0.02) [-0.04, 0.04]
repetitionc × income	0.02 (0.02) [-0.03, 0.06]
repetitionc × aot	-0.03 (0.02) [-0.08, 0.01]
repetitionc × covid_concern	0.02 (0.02) [-0.02, 0.06]
repetitionc × conspiracy	0.00 (0.02) [-0.05, 0.04]
conditiond × age	0.00 (0.05) [-0.11, 0.10]
conditiond × gender	-0.06 (0.05) [-0.17, 0.04]
conditiond × education	0.03 (0.06) [-0.08, 0.15]
conditiond × income	-0.02 (0.06) [-0.14, 0.09]
conditiond × aot	-0.01 (0.06) [-0.12, 0.10]
conditiond × covid_concern	-0.12 (0.06) [-0.23, -0.01]
conditiond × conspiracy	-0.09 (0.05) [-0.19, 0.02]
repetitionc × conditiond × age	0.02 (0.03) [-0.04, 0.08]
repetitionc × conditiond × gender	0.03 (0.03) [-0.03, 0.09]
repetitionc × conditiond × education	0.00 (0.03) [-0.07, 0.06]
repetitionc × conditiond × income	0.00 (0.03) [-0.06, 0.07]
repetitionc × conditiond × aot	0.00 (0.03) [-0.06, 0.06]
repetitionc × conditiond × covid_concern	-0.06 (0.03) [-0.12, 0.00]
repetitionc × conditiond × conspiracy	-0.01 (0.03) [-0.07, 0.05]

Standard deviations and 95% intervals of posterior distributions are shown. Only false headlines are included.

Supplementary Table 4. India experiment (only control group).

Parameter	Estimate
Intercept	4.01 (0.05) [3.90, 4.11]
veracityc	0.33 (0.07) [0.19, 0.48]
repetitionc	0.12 (0.02) [0.08, 0.17]
age	-0.13 (0.04) [-0.20, -0.06]
gender	0.02 (0.03) [-0.05, 0.09]
education	0.07 (0.04) [0.00, 0.14]
income	0.05 (0.03) [-0.01, 0.12]
aot	-0.38 (0.04) [-0.45, -0.30]
covid_concern	0.10 (0.03) [0.04, 0.17]
conspiracy	0.13 (0.04) [0.06, 0.20]
veracityc × repetitionc	0.01 (0.04) [-0.08, 0.10]
veracityc × age	0.05 (0.03) [0.00, 0.10]
veracityc × gender	-0.02 (0.02) [-0.07, 0.03]
veracityc × education	0.02 (0.03) [-0.03, 0.07]
veracityc × income	0.04 (0.02) [-0.01, 0.08]
veracityc × aot	0.18 (0.03) [0.13, 0.23]
veracityc × covid_concern	0.01 (0.02) [-0.04, 0.05]
veracityc × conspiracy	0.04 (0.02) [0.00, 0.09]
repetitionc × age	0.02 (0.02) [-0.02, 0.06]
repetitionc × gender	0.01 (0.02) [-0.03, 0.04]
repetitionc × education	0.02 (0.02) [-0.02, 0.06]
repetitionc × income	-0.04 (0.02) [-0.07, 0.00]
repetitionc × aot	0.02 (0.02) [-0.02, 0.06]
repetitionc × covid_concern	0.02 (0.02) [-0.02, 0.05]
repetitionc × conspiracy	0.03 (0.02) [-0.01, 0.06]
veracityc × repetitionc × age	-0.03 (0.03) [-0.10, 0.04]
veracityc × repetitionc × gender	-0.02 (0.03) [-0.09, 0.04]
veracityc × repetitionc × education	-0.06 (0.04) [-0.13, 0.01]
veracityc × repetitionc × income	0.04 (0.03) [-0.03, 0.10]
veracityc × repetitionc × aot	0.02 (0.04) [-0.05, 0.09]
veracityc × repetitionc × covid_concern	-0.03 (0.03) [-0.09, 0.03]
veracityc × repetitionc × conspiracy	0.01 (0.03) [-0.05, 0.08]

Standard deviations and 95% intervals of posterior distributions are shown.

Supplementary Table 5. Philippines experiment (only control group)

Parameter	Estimate
Intercept	3.95 (0.06) [3.83, 4.07]
veracityc	0.40 (0.10) [0.20, 0.59]
repetitionc	0.10 (0.02) [0.06, 0.14]
age	-0.09 (0.03) [-0.15, -0.03]
gender	0.07 (0.03) [0.01, 0.13]
education	-0.06 (0.03) [-0.13, 0.00]
income	0.03 (0.03) [-0.04, 0.10]
aot	-0.34 (0.03) [-0.40, -0.27]
covid_concern	0.08 (0.03) [0.02, 0.14]
conspiracy	0.16 (0.03) [0.10, 0.22]
veracityc × repetitionc	-0.04 (0.04) [-0.12, 0.03]
veracityc × age	0.02 (0.03) [-0.03, 0.07]
veracityc × gender	-0.09 (0.03) [-0.14, -0.04]
veracityc × education	0.06 (0.03) [0.00, 0.11]
veracityc × income	0.04 (0.03) [-0.01, 0.10]
veracityc × aot	0.23 (0.03) [0.17, 0.28]
veracityc × covid_concern	0.05 (0.03) [0.00, 0.10]
veracityc × conspiracy	-0.09 (0.03) [-0.14, -0.04]
repetitionc × age	-0.01 (0.02) [-0.04, 0.02]
repetitionc × gender	0.03 (0.02) [0.00, 0.06]
repetitionc × education	-0.02 (0.02) [-0.05, 0.02]
repetitionc × income	0.02 (0.02) [-0.01, 0.05]
repetitionc × aot	-0.03 (0.02) [-0.06, 0.00]
repetitionc × covid_concern	0.00 (0.02) [-0.03, 0.03]
repetitionc × conspiracy	-0.01 (0.02) [-0.05, 0.02]
veracityc × repetitionc × age	0.01 (0.03) [-0.05, 0.06]
veracityc × repetitionc × gender	0.04 (0.03) [-0.02, 0.09]
veracityc × repetitionc × education	-0.03 (0.03) [-0.09, 0.02]
veracityc × repetitionc × income	0.01 (0.03) [-0.05, 0.07]
veracityc × repetitionc × aot	0.01 (0.03) [-0.05, 0.07]
veracityc × repetitionc × covid_concern	-0.04 (0.03) [-0.09, 0.01]
veracityc × repetitionc × conspiracy	-0.02 (0.03) [-0.07, 0.03]

Standard deviations and 95% intervals of posterior distributions are shown.

Supplementary Table 6. Content moderator experiment 2 (control and treatment groups)

Parameter	Estimate
Intercept	3.31 (0.11) [3.09, 3.53]
repetitionC	0.10 (0.05) [0.00, 0.20]
conditionD	-0.23 (0.15) [-0.53, 0.06]
ageZ	0.01 (0.09) [-0.16, 0.18]
genderZ	0.06 (0.10) [-0.13, 0.26]
aotZ	-0.43 (0.10) [-0.63, -0.23]
covidconcernZ	-0.04 (0.09) [-0.21, 0.14]
sesZ	0.11 (0.09) [-0.07, 0.29]
attentionZ	-0.04 (0.10) [-0.24, 0.16]
educationZ	-0.01 (0.09) [-0.20, 0.17]
godZ	-0.15 (0.09) [-0.33, 0.03]
hireZ	-0.11 (0.10) [-0.32, 0.09]
repetitionC × conditionD	-0.19 (0.08) [-0.35, -0.03]
repetitionC × ageZ	-0.05 (0.05) [-0.15, 0.04]
repetitionC × genderZ	0.04 (0.05) [-0.07, 0.14]
repetitionC × aotZ	-0.07 (0.05) [-0.17, 0.04]
repetitionC × covidconcernZ	0.01 (0.05) [-0.09, 0.10]
repetitionC × sesZ	-0.07 (0.05) [-0.17, 0.03]
repetitionC × attentionZ	0.04 (0.05) [-0.07, 0.14]
repetitionC × educationZ	-0.01 (0.05) [-0.11, 0.08]
repetitionC × godZ	-0.04 (0.05) [-0.14, 0.06]
repetitionC × hireZ	-0.03 (0.05) [-0.14, 0.07]
conditionD × ageZ	-0.07 (0.15) [-0.36, 0.22]
conditionD × genderZ	-0.11 (0.16) [-0.42, 0.19]
conditionD × aotZ	0.15 (0.16) [-0.15, 0.46]
conditionD × covidconcernZ	-0.17 (0.19) [-0.53, 0.20]
conditionD × sesZ	-0.07 (0.16) [-0.39, 0.25]
conditionD × attentionZ	0.06 (0.15) [-0.24, 0.36]
conditionD × educationZ	-0.13 (0.15) [-0.43, 0.17]
conditionD × godZ	0.26 (0.14) [-0.01, 0.53]
conditionD × hireZ	-0.07 (0.15) [-0.38, 0.23]
repetitionC × conditionD × ageZ	0.11 (0.08) [-0.05, 0.27]
repetitionC × conditionD × genderZ	-0.18 (0.08) [-0.34, -0.02]
repetitionC × conditionD × aotZ	-0.14 (0.08) [-0.30, 0.02]
repetitionC × conditionD × covidconcernZ	0.02 (0.10) [-0.17, 0.22]
repetitionC × conditionD × sesZ	-0.05 (0.09) [-0.22, 0.12]
repetitionC × conditionD × attentionZ	-0.10 (0.08) [-0.26, 0.06]
repetitionC × conditionD × educationZ	-0.06 (0.08) [-0.21, 0.10]
repetitionC × conditionD × godZ	0.06 (0.08) [-0.08, 0.21]
repetitionC × conditionD × hireZ	-0.06 (0.08) [-0.22, 0.10]

Standard deviations and 95% intervals of posterior distributions are shown. Only false headlines are included.

Supplementary Table 7. Content moderator experiment 2 (only control group)

Parameter	Estimate
Intercept	3.53 (0.08) [3.37, 3.68]
veracityC	0.44 (0.10) [0.25, 0.62]
repetitionC	0.08 (0.04) [0.01, 0.15]
ageZ	-0.01 (0.06) [-0.13, 0.12]
genderZ	0.11 (0.07) [-0.03, 0.26]
aotZ	-0.33 (0.08) [-0.48, -0.17]
covidconcernZ	0.01 (0.07) [-0.12, 0.15]
sesZ	0.10 (0.07) [-0.04, 0.23]
attentionZ	-0.04 (0.07) [-0.18, 0.11]
educationZ	-0.03 (0.07) [-0.16, 0.11]
godZ	-0.14 (0.07) [-0.27, -0.01]
hireZ	-0.11 (0.08) [-0.27, 0.04]
veracityC × repetitionC	-0.02 (0.07) [-0.17, 0.12]
veracityC × ageZ	-0.04 (0.06) [-0.16, 0.08]
veracityC × genderZ	0.11 (0.07) [-0.03, 0.24]
veracityC × aotZ	0.21 (0.07) [0.07, 0.35]
veracityC × covidconcernZ	0.11 (0.06) [-0.01, 0.23]
veracityC × sesZ	-0.04 (0.06) [-0.17, 0.09]
veracityC × attentionZ	0.01 (0.07) [-0.13, 0.14]
veracityC × educationZ	-0.03 (0.06) [-0.16, 0.10]
veracityC × godZ	0.03 (0.06) [-0.10, 0.15]
veracityC × hireZ	0.00 (0.07) [-0.14, 0.14]
repetitionC × ageZ	-0.07 (0.03) [-0.14, -0.01]
repetitionC × genderZ	0.07 (0.03) [0.00, 0.13]
repetitionC × aotZ	-0.03 (0.03) [-0.10, 0.04]
repetitionC × covidconcernZ	-0.01 (0.03) [-0.07, 0.05]
repetitionC × sesZ	-0.06 (0.03) [-0.12, 0.00]
repetitionC × attentionZ	0.03 (0.04) [-0.03, 0.10]
repetitionC × educationZ	0.00 (0.03) [-0.06, 0.07]
repetitionC × godZ	0.01 (0.03) [-0.06, 0.07]
repetitionC × hireZ	-0.05 (0.04) [-0.12, 0.03]
veracityC × repetitionC × ageZ	-0.03 (0.07) [-0.16, 0.10]
veracityC × repetitionC × genderZ	0.05 (0.07) [-0.09, 0.19]
veracityC × repetitionC × aotZ	0.07 (0.07) [-0.07, 0.22]
veracityC × repetitionC × covidconcernZ	-0.04 (0.06) [-0.16, 0.09]
veracityC × repetitionC × sesZ	-0.01 (0.07) [-0.14, 0.13]
veracityC × repetitionC × attentionZ	0.02 (0.07) [-0.12, 0.17]
veracityC × repetitionC × educationZ	0.02 (0.07) [-0.11, 0.15]
veracityC × repetitionC × godZ	0.08 (0.07) [-0.06, 0.21]
veracityC × repetitionC × hireZ	-0.01 (0.08) [-0.16, 0.14]

Standard deviations and 95% intervals of posterior distributions are shown.
